# Novel Insights into the Role of circRNAs in Cancer Immunotherapy Resistance and Clinical Implications

**DOI:** 10.3390/ijms27083678

**Published:** 2026-04-20

**Authors:** Kangdi Yang, Yu Zhang, Junjie Xiong, Bin Ai, Dan Han, Xiaodan Chong

**Affiliations:** 1 Clinical Oncology Institute, Translational Medicine Center, Naval Medical University, Shanghai 200433, China; ykd970214@163.com (K.Y.); zhangyu20250417@163.com (Y.Z.); 2 School of Life Sciences, East China Normal University, Shanghai 200241, China; ecnujeuoxjj@163.com; 3 Department of Precision Medicine, Translational Medicine Center, Naval Medical University, Shanghai 200433, China; aibin@smmu.edu.cn; 4 School of Pharmacy, Naval Medical University, Shanghai 200433, China

**Keywords:** circRNA, immunotherapy resistance, tumor, immune escape, signaling pathway

## Abstract

Cancer therapies are increasingly reliant on immunotherapeutic interventions; however, the persistent emergence of primary, adaptive, and acquired resistance severely limits durable clinical efficacy. Circular RNAs (circRNAs), distinguished by their extreme structural stability and covalently closed loops, have recently been established as potent orchestrators of this immune evasion. This review systematically synthesizes current advancements detailing how circRNAs undermine anti-tumor immunity across diverse malignancies. Specifically, we delineate their critical roles in post-transcriptionally upregulating immune checkpoint molecules (e.g., PD-L1), mediating intercellular immunosuppression via exosomal transfer, and metabolically reprogramming the tumor microenvironment to drive CD8+ T-cell exhaustion and macrophage polarization. Ultimately, we conclude that translating these molecular insights into clinical practice is paramount. Beyond serving as predictive biomarkers, engineering circRNA-targeted therapies and exploiting tumor-specific circRNAs to develop novel anti-tumor vaccines represent essential, paradigm-shifting strategies to definitively overcome immune checkpoint inhibitor resistance.

## 1. Introduction

Cancer has become one of the leading causes of death worldwide and is characterized by genetic imbalance, which also underlies the characteristics of tumors [1]. In clinical practice, in addition to surgical treatment, there are also chemotherapy, radiotherapy, and immunotherapy [2]. Although there are many ways to treat it, it is still difficult to achieve ideal results in clinical practice. Among them, the development of tumor drug resistance during treatment is one of the main causes of cancer-related deaths worldwide [3]. This may be due to the inability of tumor cells to respond to conventional therapies such as chemotherapy, immunotherapy and radiotherapy through multiple molecular mechanisms, and the activation of these therapies is difficult to predict, prevent or reverse [4].

Among the various treatments for tumors, tumor immunotherapy can make a targeted drug applicable to different cancers, even advanced metastatic solid tumors [5]. The success of PD-1 and PD-L1, for example, is clinically applicable to most cancer types. In addition, CAR-T and CAR-NK, which modify immune cells, also have excellent therapeutic effects in clinical practice [6]. However, the problem of drug resistance will also arise, resulting in failure to achieve the desired therapeutic effect.

Drug resistance mainly refers to the tolerance of tumor cells to anti-tumor drugs. Once tolerance is developed, the therapeutic effect will be significantly reduced. Clinically, immune evasion presents through three distinct classifications: primary, adaptive, and acquired resistance. Primary resistance denotes malignancies inherently refractory to initial therapeutic intervention, typically due to poor baseline antigenicity [7]. Adaptive resistance occurs when the tumor dynamically alters its microenvironment to shield itself from an initially active immune assault [8]. Finally, acquired resistance characterizes the subsequent relapse of initially responsive tumor cell populations following prolonged therapeutic exposure [9].

The occurrence and development of tumor immunotherapy resistance, through epigenetic changes, signal pathway regulation and other mechanisms, changes the expression of its own proteins, destroys the immune recognition mechanism, and evades immunotherapy, thereby producing tolerance to immunotherapy drugs [10]. Hence, it is important to reduce the probability of drug resistance as much as possible, through methods such as multidrug combinations, in order to kill as many cancer cells as possible and reduce the probability of drug resistance of tumor cells [11]. Therefore, the selection of more comprehensive and effective gene detection technology can better avoid the drug resistance caused by tumor heterogeneity in clinical practice. For example, the current advanced circulating tumor DNA detection (ctDNA) captures the DNA released by cancer cells in the blood to obtain cellular and genetic information at different tumor sites and drug-resistant mutations [12].

Despite the ability of cancer cells to survive even at high doses of combined use of one or more anticancer drugs, anti-tumor resistance develops. However, in recent decades, scientists have explored the physiology of tumors in which immunotherapy fails and leads to a resistant phenotype at the molecular and cellular levels and from many different perspectives [13].

Circular RNAs (circRNAs) play a crucial role in cancer biology, contributing to tumorigenesis and progression. These molecules are involved in regulating gene expression, impacting cell proliferation, migration, and invasion [13]. Their unique circular structure enhances stability, making circRNAs potential biomarkers for cancer diagnosis and prognosis. Understanding the significance of circRNAs in cancer sheds light on novel therapeutic targets and diagnostic strategies for more effective and personalized treatment approaches.

While previous studies have broadly outlined circRNA functions, comprehensive reviews addressing their specific mechanisms in immune checkpoint inhibitor resistance remain limited. This review bridges this gap by systematically evaluating the latest evidence (up to 2026) on how circRNAs govern exosomal intercellular communication and metabolic reprogramming to drive immunotherapy resistance. Altered expression of circRNAs has been associated with resistance to checkpoint inhibitors and other immunotherapies. Understanding the intricate interplay between circRNAs and immune regulation could provide insights into mechanisms of resistance, potentially paving the way for developing strategies to overcome immunotherapy challenges in cancer treatment.

## 2. Circular RNA and Its Function

circRNAs were initially observed in the 1970s in plant viroids and later in animal cells. While their discovery can be traced back to this early period, their functional significance and widespread presence gained increased recognition with advancements in RNA sequencing technologies. Researchers, including Sanger and colleagues, played a key role in identifying and characterizing circRNAs, revealing their unique structure and highlighting their potential roles in gene regulation. circRNAs, namely Circular RNAs, are a class of non-coding RNA molecules that do not have a 5′ terminal cap and a 3′ terminal poly (A) tail and form a circular structure by covalent bonds. circRNA is a new class of RNA that is different from traditional linear RNA [14,15]. It has a closed-loop structure and exists in a large number of eukaryotic transcriptomes. Most circRNAs are composed of exon sequences, which are conserved in different species and differentially expressed in different tissues and different developmental stages. Moreover, circular RNAs are more stable than linear RNAs because they are not sensitive to nucleases, which makes circRNAs have obvious advantages in the development and application of new clinical diagnostic markers. Compared with the traditional linear RNA, circRNA has stronger stability. And it is more than 10 times as much as the linear mRNA in quantity. Although it is highly conserved, some parts have rapid evolutionary changes. The vast majority of circRNA is derived from exons, and a small part is formed by direct looping of introns, which have a certain tissue, time sequence and disease specificity [16]. CircRNAs are robustly stable non-coding molecules due to their closed-loop structures. Rather than merely engaging in basic cellular maintenance, they serve as potent immunomodulators. Specifically, circRNAs regulate immune cell proliferation, govern macrophage polarization, and dictate the secretion profiles of inflammatory cytokines within the tumor microenvironment.

Beyond acting as microRNA sponges, circRNAs deploy sophisticated mechanisms in immunity. They frequently function as protein scaffolds, physically orchestrating or disrupting the assembly of critical immune signaling complexes. Furthermore, certain circRNAs undergo cap-independent translation to produce functional micropeptides. These translated peptides can act as cryptic antigens, effectively priming naive T-cells and stimulating targeted adaptive immune responses [13]. It is critical to note that the experimental rigor across current circRNA studies varies. While robust investigations mandate RNase R resistance assays and junction-specific Sanger sequencing to confirm RNA circularity, some foundational reports relied primarily on bioinformatics and correlative expression. Future research must universally adopt these stringent validation protocols. In addition to regulatory roles, circRNAs hold potential to influence the tumor’s antigenic profile. Peptides translated from tumor-specific circRNAs can act as potent neoantigens. Furthermore, circRNAs that modulate DNA damage repair pathways may indirectly influence the overall Tumor Mutational Burden (TMB), shaping the tumor’s inherent immunogenicity. The functional identity of certain circRNAs exhibits striking context-dependency. A specific circRNA might suppress tumor immunity in one histological subtype while demonstrating oncogenic or immune-activating properties in another, dictated largely by the divergent abundance of target miRNAs within varying cellular microenvironments. Unlike static genomic markers such as Tumor Mutational Burden (TMB) or Microsatellite Instability (MSI), which merely predict baseline neoantigen potential, circRNAs act dualistically as both dynamic predictive biomarkers and active mechanistic mediators of resistance. Despite their robust translational potential, a review of current trial registries reveals a stark absence of active, large-scale clinical trials deploying circRNA-targeted immunotherapies, highlighting an urgent frontier for clinical oncology.

**Figure 1:**
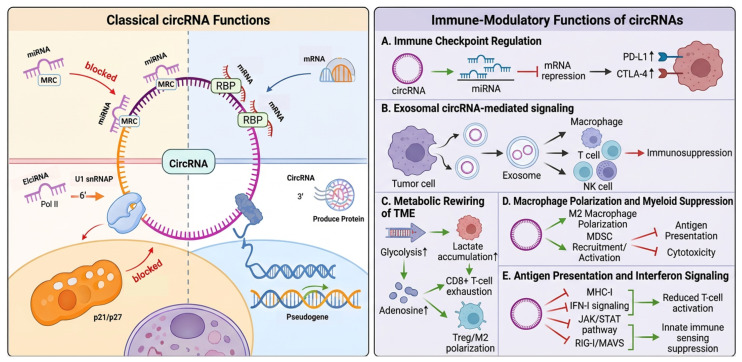
Functional diversity of circRNAs with emphasis on immune-modulatory roles. CircRNAs exert classical functions as miRNA sponges/ceRNAs, protein scaffolds or decoys, cap-independent translation templates, transcriptional regulators, and potential pseudogene-related modulators. In addition, circRNAs participate in immune regulation by controlling immune checkpoint expression, mediating exosomal transfer, reprogramming tumor metabolism, promoting macrophage polarization, and suppressing T/NK-cell activity. These immune-modulatory functions contribute to tumor immune escape and immunotherapy resistance. Abbreviations: circRNA, circular RNA; miRNA, microRNA; ceRNA, competing endogenous RNA; RBP, RNA-binding protein; TME, tumor microenvironment; PD-1, programmed cell death protein 1; PD-L1, programmed cell death ligand 1; CTLA-4, cytotoxic T-lymphocyte-associated protein 4; B7-H3, B7 homolog 3; TIM-3, T-cell immunoglobulin and mucin-domain containing-3; NK, natural killer; MDSC, myeloid-derived suppressor cell; Treg, regulatory T-cell; MHC-I, major histocompatibility complex class I; IFN-I, type I interferon; JAK/STAT, Janus kinase/signal transducer and activator of transcription; RIG-I, retinoic acid-inducible gene I; MAVS, mitochondrial antiviral-signaling protein; MRC, mitochondrial RNA complex; Pol II, RNA polymerase II; U1 snRNP, U1 small nuclear ribonucleoprotein; U2AF65, U2 auxiliary factor 65 kDa; M2, M2 macrophage.This figure was created in BioRender. Yang, C. (2026). https://BioRender.com/xi9jnke; accessed on 22 March 2026.

circRNAs are rich in functions (Figure 1). First, circRNA can act as a protein antagonist or decoy to inhibit protein activity. For example, circ-Foxo3 can interact with cell cycle-related proteins, including p21 and p27, thereby blocking the role of these proteins in cancer cell cycle progression [14,17,18]. Second, circRNAs are miRNA sponges or competing endogenous RNAs (ceRNAs). circRNAs are mainly found in the cytoplasm and rich in miRNA binding sites. They can act as miRNA sponges or ceRNAs in cells, automatically isolating or competitively inhibiting the activity of miRNAs, and then deregulating the inhibitory effect of miRNAs on their target genes, increasing the expression level of target genes [19,20]. Third, they can be used as a translation template. Although most circRNAs do not have the ability to bind to the ribosome for translation, research data show that a small number of endogenous circRNAs can be translated into proteins or peptides by N6-methyladenosine modification or internal ribosome entry site (IRES) [21]. Fourth, pseudogenes can be generated. Pseudogenes are mainly derived from reverse transcription of linear mRNAs. A large number of pseudogenes derived from circRNAs have been identified by examining the backsplicing junction sequences of the genome [22]. Fifth, they can regulate gene transcription. Most circRNAs in the cytoplasm are derived from exons, while intronic circRNAs are mainly located in the nucleus and play a role in transcriptional regulation [23]. In addition, circRNAs were shown to interact with Pol II transcription compounds to activate transcription of their parent genes [24]. The dysregulation of classical kinase cascades, such as the MAPK and PI3K/AKT pathways, is frequently upstream-regulated by specific circRNAs. By sponging suppressive miRNAs, oncogenic circRNAs derepress key target genes, thereby continuously activating WNT/β-catenin and PI3K signaling networks to drive immune evasion. Recent evidence suggests that circRNA plays an important role in regulating tumor immunotherapy [25]. Among cancer vaccines, RNA cancer vaccines are considered to be the most promising ones, which can rapidly express antigens in the cytoplasm and generate a strong immune response because circRNA is not easily degradation by exonuclease [26]. Since circRNA is more stable than linear mRNA, it can be used to replace therapeutic proteins and peptides in cancer immunotherapy.

## 3. Immunotherapy in Tumors

**Figure 2:**
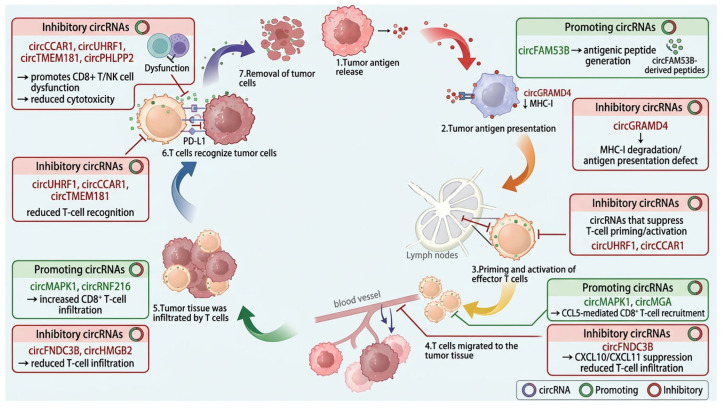
circRNAs regulate distinct steps of the tumor–immune cycle. The tumor–immune cycle includes tumor antigen release, antigen presentation, priming and activation of effector T-cells, T-cell migration to tumor tissue, T-cell infiltration, tumor cell recognition, and tumor cell killing. CircRNAs can modulate each step by regulating antigenicity, antigen presentation, chemokine signaling, T-cell recruitment, checkpoint expression, and cytotoxic effector function. Representative circRNAs are shown as examples of circRNA-mediated enhancement or suppression of specific cycle steps. Abbreviations: circRNA, circular RNA; DC, dendritic cell; TCR, T-cell receptor; MHC-I, major histocompatibility complex class I; NK, natural killer; PD-1, programmed cell death protein 1; PD-L1, programmed cell death ligand 1; CCL5, C-C motif chemokine ligand 5; CXCL10, C-X-C motif chemokine ligand 10; CXCL11, C-X-C motif chemokine ligand 11; IFN-γ, interferon-gamma. This figure was created in BioRender. Yang, C. (2026) https://BioRender.com/xi9jnke; accessed on 22 March 2026.

Cancer immunotherapy aims to restore anti-tumor immunity by reactivating the tumor–immune cycle and overcoming immune tolerance within the tumor microenvironment [27]. As shown in Figure 2, The tumor–immune cycle comprises seven sequential steps: tumor antigen release, tumor antigen presentation, priming and activation of effector T-cells, T-cell migration to tumor tissue, T-cell infiltration, recognition of tumor cells, and tumor cell killing [28,29]. Disruption of any step can weaken the anti-tumor response and facilitate immune escape [30,31].

At present, the main immunotherapeutic approaches include monoclonal anti-body-based checkpoint blockade, therapeutic antibodies, cancer vaccines, cell therapy, small-molecule inhibitors, adoptive cell therapy, and oncolytic virus therapy [32,33,34,35,36,37,38,39,40,41,42,43,44,45]. Among these, immune checkpoint inhibitors are the most clinically developed and include antibodies targeting PD-1, PD-L1, CTLA-4, LAG-3, TIM-3, TIGIT, and VISTA [34,45,46,47,48,49]. Monoclonal antibodies can directly bind tumor antigens or regulate cell signaling to inhibit proliferation and promote apoptosis, as exemplified by rituximab and trastuzumab [39,40,41,42]. Adoptive cell therapies such as CAR-T and CAR-NK have shown strong activity in hematological malignancies and are increasingly being evaluated for solid tumors [17,50,51,52]. Oncolytic virus therapy and tumor vaccines represent additional strategies to stimulate anti-tumor immunity and broaden the therapeutic repertoire [37,53,54,55]. Other cell-based or biologic therapies, including stem cell- and immune cell-based approaches, are also being explored in advanced disease settings [45,56,57,58]. Collectively, these modalities seek to restore effective anti-tumor immune responses, but their efficacy remains limited by resistance mechanisms operating at both the tumor cell and microenvironmental levels [27,59].

Because circRNAs can influence antigen presentation, checkpoint signaling, cytokine production, and immune-cell recruitment, they are increasingly recognized as important regulators of immunotherapy response. Accordingly, the tumor–immune cycle provides a useful framework for understanding circRNA-mediated immune modulation and resistance.

## 4. The Challenge of Immunotherapy: Drug Resistance Emergence

Despite major clinical advances, resistance remains a central obstacle to durable immunotherapy responses [60,61]. Tumor-intrinsic resistance mechanisms often involve activation of oncogenic pathways, altered antigenicity, and disruption of immune recognition. For example, MAPK pathway activation and PI3K signaling can promote VEGF and IL-8 production, thereby limiting T-cell recruitment [62]. Aberrant PI3K/AKT signaling and PTEN loss are also associated with reduced IFN-γ, granzyme B, and tumor-infiltrating T-cell abundance [63]. Sustained WNT/β-catenin signaling contributes to T-cell exclusion, and high β-catenin expression has been linked to T-cell–poor melanoma [64,65,66,67,68]. In addition, PD-L1 overexpression is driven by PTEN loss.

PI3K/AKT mutations, EGFR mutations, MYC overexpression, and CDK5-mediated PD-L1 3′-UTR truncation, while defective IFN-γ signaling further impairs immune responses [69,70]. Mutations or deletions in IFN-γ pathway components, including IFN-γ receptors, JAK1/JAK2, STATs, and IRF1, are also associated with resistance to checkpoint blockade [71,72,73,74,75,76]. Loss of tumor antigens or defects in antigen presentation, including abnormalities in proteasome components, transporters, MHC molecules, and β-2-microglobulin (B2M), further undermine immune recognition [77,78,79,80,81,82].

Tumor-extrinsic resistance is largely shaped by the immunosuppressive tumor microenvironment, which contains regulatory T-cells, myeloid-derived suppressor cells, tumor-associated macrophages, inhibitory checkpoints, and suppressive cytokines [83,84,85,86,87,88,89,90,91,92,93,94]. Tregs suppress effector T-cell activity through inhibitory cytokines such as IL-10, IL-35, and TGF-β or through direct cell–cell contact [83,84,85]. MDSCs promote angiogenesis, invasion, and metastasis and are associated with poor prognosis and reduced checkpoint inhibitor efficacy [86,87,88,89]. M2 macrophages, which dominate TAM populations in many tumors, secrete IL-10 and TGF-β to suppress immune responses and favor tumor progression [89,90]. Chemokines and chemokine receptors further recruit suppressive immune cells into the tumor microenvironment [91,92,93,94]. CD28 expression on tumor-infiltrating cells also influences PD-1 reactivation and may serve as a predictor of anti-PD-1 response [95].

## 5. CircRNA in Drug Resistance of Tumor Immunotherapy

To explicitly highlight current research trends, a quantitative analysis of the 77 immune-regulatory circRNAs discussed herein was conducted. The data reveals that lung (21 circRNAs, [96,97,98,99,100,101,102,103,104,105,106,107,108,109,110,111,112,113,114,115,116]), gastrointestinal (19 circRNAs, [117,118,119,120,121,122,123,124,125,126,127,128,129,130,131,132,133,134,135]), and hepatic tumors (16 circRNAs, [136,137,138,139,140,141,142,143,144,145,146,147,148,149,150,151]) represent the primary focal points of circRNA-mediated immunotherapy resistance.

**Figure 3:**
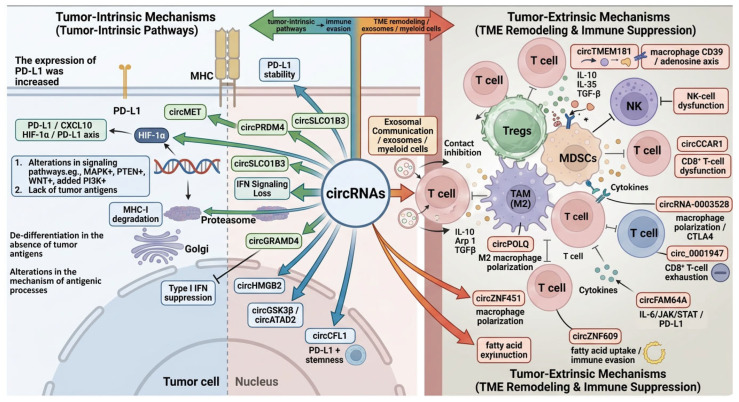
circRNA-mediated internal and external mechanisms of tumor immunotherapy resistance. CircRNAs promote immunotherapy resistance through tumor-intrinsic mechanisms, including immune checkpoint upregulation, antigen-presentation defects, loss of interferon signaling, and metabolic rewiring, as well as tumor-extrinsic mechanisms involving exosomal communication, macrophage polarization, MDSC recruitment, Treg expansion, and NK/T-cell dysfunction. Representative circRNAs are placed within the corresponding regulatory modules to illustrate how circRNAs integrate cell-intrinsic and microenvironmental pathways to promote immune escape. Abbreviations: circRNA, circular RNA; TME, tumor microenvironment; PD-1, programmed cell death protein 1; PD-L1, programmed cell death ligand 1; CTLA-4, cytotoxic T-lymphocyte-associated protein 4; B7-H3, B7 homolog 3; TIM-3, T-cell immunoglobulin and mucin-domain containing-3; MDSC, myeloid-derived suppressor cell; Treg, regulatory T-cell; TAM, tumor-associated macrophage; NK, natural killer; MHC-I, major histocompatibility complex class I; IFN-I, type I interferon; JAK/STAT, Janus kinase/signal transducer and activator of transcription; HIF-1α, hypoxia-inducible factor 1-alpha; RIG-I, retinoic acid-inducible gene I; MAVS, mitochondrial antiviral-signaling protein; WNT, Wingless-related integration site; PI3K, phosphoinositide 3-kinase; PTEN, phosphatase and tensin homolog; MAPK, mitogen-activated protein kinase; Arg1, arginase 1. This figure was created in BioRender. Yang, C. (2026) https://BioRender.com/xi9jnke; accessed on 22 March 2026.

In recent years, a new class of lncRNAs, named circRNAs, has been widely studied in cancer biology. circRNAs are extracted from pre-mRNA by a noncanonical alternative splicing event called backsplicing. This process imbues circRNAs with a unique fundamental structural feature that differs from the common linear lncRNAs, namely a covalent loop continuous loop structure without a deadenylate terminus. circRNAs are very stable because their unique structure is resistant to exonuclease-mediated degradation [18,19] (Figure 3).

A large number of different circRNAs have been demonstrated in inhibiting or activating target genes involved in drug-induced apoptosis, thereby triggering aberrant and atypical responses to commonly used chemotherapy regimens [152]. Overexpression of “tumor suppressor” circRNAs supports the highest sensitivity, whereas “oncogenic” circRNAs can promote chemotherapy resistance by acting as mediators of MDR [153]. circRNA can directly or indirectly regulate various pathways related to chemotherapy resistance, such as those involved in altering drug efflux, inhibiting apoptotic pathways, CSC enrichment, and promoting EMT and angiogenesis [154]. Many studies have further shown that circRNA can promote the enhancement of DNA repair in tumor cells through a variety of mechanisms, including positive regulation of transcription factors that respond to DNA damage, inhibition of factors that hinder DNA repair, and interaction with DNA repair proteins and miRNAs, thereby inhibiting anti-tumor drug function [155] by acting as a repair mediator at sites of DNA damage. On the other hand, circRNA may inhibit tumor cell apoptosis and hinder the apoptotic function of anti-tumor drugs by controlling related signal transduction pathways, such as Wnt/β-catenin and PI3K/AKT cascades, and by interacting with relevant miRNAs [70]. In this context, the following sections involve circRNAs, which have been reported to regulate tumor response to conventional chemotherapy through multiple mechanisms, depending on drug class and function [156]. Not only that, circRNA also plays an important role in immunotherapy resistance.

Immune checkpoint drugs are a class of immunosuppressive molecules that can regulate the strength and breadth of the immune response, thereby avoiding the damage and destruction of normal tissues. In the process of tumor occurrence and development, immune checkpoint has become one of the main reasons for immune tolerance. Immune checkpoint therapy is a therapeutic method to kill tumor cells by regulating the activity of T-cells through a series of pathways such as co-inhibition or co-stimulation signals. At present, immune checkpoint drugs are mainly divided into two categories. One is PD-1 inhibitors. The other category is activators, which are currently under clinical investigation. However, the clinical research on immune checkpoint inhibitors is the most mature and full, and it is also widely used.

At present, the main immune checkpoint inhibitors include CTLA-4 antibody, PD-1 antibody, LAG-3 antibody and TIM-3 antibody, TIGIT antibody, VISTA antibody, etc. [29]. The clinical success of immune checkpoint inhibitors has enabled the immunotherapy of cancer to play a role in traditional Chinese medicine. However, how to improve the efficiency of immune checkpoint blockade and how to use combination therapy of immune checkpoint inhibitors to achieve durable anti-tumor responses in patients who cannot be treated with immune checkpoint inhibitors alone are urgent problems to be solved. Not only that, immune checkpoint inhibitor resistance will also lead to low response rate and tumor recurrence. Among the causes of drug resistance, circRNA plays a more important role in regulating immune checkpoint inhibitor resistance.

In summary, circRNA plays an important role in tumor immunotherapy and has a strong research value in immune checkpoint inhibitors, because tumor cells generally lead to the upregulation of immune checkpoints, increasing the difficulty of treatment, and even producing drug resistance. Therefore, circRNA is also very valuable for research and exploration in the drug resistance of tumor immunotherapy.

### 5.1. Circular RNA’s Role in Immunotherapy Resistance in Hepatic Tumors

In hepatic tumors, circRNAs regulate immunotherapy resistance through several recurrent and convergent pathways rather than isolated single-gene effects. One major theme is the circRNA–miRNA–immune checkpoint axis, in which circRNAs promote immune escape by reinforcing PD-L1-related signaling or other checkpoint pathways. For example, exosomal circTMEM181 is elevated in hepatocellular carcinoma (HCC) patients with poor response to anti-PD-1 therapy and poor postoperative prognosis. Mechanistically, circTMEM181 sponges miR-488-3p and upregulates CD39 in macrophages, thereby supporting an adenosine-rich immunosuppressive microenvironment and conferring anti-PD-1 resistance [157] (Table 1). Similarly, exosomal circUHRF1 is associated with NK-cell dysfunction in HCC; it suppresses NK-cell-derived IFN-γ and TNF-α secretion and increases TIM-3 expression by degrading miR-449c-5p, ultimately contributing to anti-PD-1 resistance [158] (Table 1). circMET (hsa_circ_0082002) also promotes HCC progression and immunosuppression through the miR-30-5p/Snail/DPP4/CXCL10 axis [159] (Table 1), while circSOD2 enhances HCC progression and immune evasion by sponging miR-497-5p and upregulating ANXA11 [160] (Table 1).

A second prominent theme is myeloid-cell programming and exosome-mediated immune remodeling. circRNA-0003528 promotes macrophage polarization by downregulating miR-224-5p, miR-324-5p, and miR-488-5p and thereby increasing CTLA4 expression [161] (Table 1). Exosomal circCCAR1, released from HCC cells, promotes CD8+ T-cell dysfunction and anti-PD-1 resistance through the miR-127-5p/WTAP axis [162] (Table 1). These findings suggest that circRNAs can operate not only within tumor cells but also across immune compartments, shaping the tumor microenvironment at multiple levels.

CircRNAs also contribute to metabolic rewiring and hypoxia-associated immune escape. circPETH-147aa, a circRNA-derived protein, is transferred from tumor-associated macrophages to HCC cells via extracellular vesicles, where it enhances glycolysis and metastasis. It weakens anti-HCC immunity by stabilizing HuR-dependent SLC43A2 mRNA and causing methionine and leucine deficiency in cytotoxic CD8+ T-cells; notably, norathyriol can target the MEG pocket of circPETH-147aa and restore anti-PD-1 activity [163] (Table 1). circCCNY binds HSP60 and promotes its degradation via SMURF1, which in turn inactivates the MAPK pathway and enhances lenvatinib sensitivity while also reducing immune evasion through suppression of MAPK/c-Myc/PD-L1 signaling [164] (Table 1). circCDYL stabilizes hornerin by blocking SYVN1-mediated ubiquitination, thereby promoting stemness and PD-L1+ exosome-mediated immunotherapy resistance [165] (Table 1). In addition, circ0027791 promotes HCC growth and immune evasion in an m6A-dependent manner via the miR-496/PD-L1 axis [166] (Table 1). circ-0044539 enhances CXCR4 expression and promotes exosomal miR-29a-3p release under hypoxia, which increases PMN-MDSC immunosuppressive activity and supports lymph node metastasis [167] (Table 1).

A further layer of regulation involves stromal crosstalk and antigen-exclusion programs. Hepatic stellate cell-derived exosomal circWDR25 regulates ALOX15 by sponging miR-4474-3p and induces epithelial–mesenchymal transition in liver cancer cells; it also promotes CTLA-4 in hepatic stellate cells and PD-L1 in HCC cells, suggesting a role in checkpoint-associated resistance [168] (Table 1). In intrahepatic cholangiocarcinoma (ICC), exosomal circ-PTPN22 and circ-ADAMTS6 are associated with T-cell exhaustion and neutrophil extracellular trap damage, indicating that circulating circRNAs may also reflect immune dysfunction in the tumor microenvironment [169] (Table 1). Hypoxia-associated circPRDM4 promotes PD-L1 expression by recruiting HIF-1α to the CD274 promoter and inhibits CD8+ T-cell infiltration, thereby contributing to immune escape and anti-PD-1 resistance [170] (Table 1). circHMGCS1-016 participates in ICC progression and immune tolerance through the miR-1236-3p/CD73/GAL-8 axis [171] (Table 1), whereas circSLCO1B3 is stabilized by METTL3-mediated m6A methylation and promotes ICC progression and immune escape through the miR-502-5p/HOXC8/SMAD3 axis while preventing PD-L1 degradation via SPOP [172] (Table 1).

Overall, hepatic tumor-associated circRNAs converge on a restricted set of downstream nodes, including PD-L1, CD39/CD73-adenosine signaling, HIF-1α, MAPK, CXCL10/CXCR4, and macrophage/T-cell dysfunction. This convergence suggests substantial mechanistic redundancy, as multiple circRNAs may produce similar immune phenotypes through different upstream routes. From a translational perspective, circRNAs with strong tumor specificity, extracellular stability, or a non-redundant position in the resistance network may be more useful as biomarkers or combination-therapy targets than as stand-alone therapeutic candidates.

**Table 1:** The target and mechanism of circular RNA in immune therapy resistance of hepatic carcinoma.

circRNA	Cancer Type	Immune Checkpoints	Altered Mechanism	Molecular Targets	Study Model	Drug	References
circTMEM181	HCC	PD-L1	Exosome Upscale and ATP–Adenosine Pathway Inhibition.	miR-488–3p	In vitro, clinical	Sintilimab	[157]
circUHRF1	HCC	TIM-3	Inhibition of NK-cell IFN-γ and TNF-α secretion.	miR-449c–5p	In vitro, in vivo, clinical	TSR-022	[158]
circMET	HCC	PD-L1	miR-30-5p/Snail/DPP4/CXCL10 axis.	miR-30–5p	In vitro, in vivo, clinical	Sintilimab	[159]
circSOD2	HCC	PD-1	ANXA11 upregulation induced by interaction with miR-497-5p.	miR-497-5p	In vitro, in vivo	Sintilimab	[160]
circ-PTPN22	ICC	/	T-cell depletion and neutrophil extracellular trap injury.	/	In vitro, clinical	/	[161]
circPRDM4	HCC	PD-1	Hypoxia promotes HIF-1 recruitment to the CD274 promoter.	/	In vitro, in vivo, clinical		[162]
circWDR25	HCC	CTLA-4/PD-1	circWDR25/miR-4474–3p/ALOX15 axis promoted HCC cell proliferation and invasion.	miR-4474–3p	In vitro, clinical	/	[163]
circRNA-0003528	HCC	CTLA4	Macrophage polarization.	miR-224–5p, miR-324–5p, miR-488–5p	In vitro		[164]
circCCAR1	HCC	PD-L1	CD8^+^ T-cell dysfunction leads to immunosuppression.	miR-127–5p	In vitro, in vivo, clinical		[165]
CircHMGCSI-016	ICC	PD-1	Key role in ICC development and immune tolerance.	miR-1236-3p	In vitro, clinical	Sintilimab	[166]
circPETH-147aa	HCC	PD-1	Methionine and leucine insufficiency in cytotoxic CD8^+^ T-cells.	SLC43A2	In vitro, in vivo	Norathyriol	[167]
circCCNY	HCC	PD-1	Inhibition of the MAPK/c-Myc/PD-L1 signaling pathway.	/	In vitro, in vivo, clinical	/	[168]
circCDYL	HCC	PD-L1	Hornerin stabilization via blocking synoviolin 1 ubiquitination facilitates PD-L1(+) exosomes.	HRNR	In vitro, in vivo, clinical	/	[169]
Circ0027791	HCC	PD-L1	Circ0027791/miR-496/PD-L1 axis promoted facilitates HCC progression and immune evasion.	miR-496	In vitro, in vivo	/	[170]
circ0044539	HCC	/	Downregulation of Hbp1 in PMN-MDSCs.	miR-29a-3p	In vitro, in vivo, clinical	/	[171]
circSLCO1B3	ICC	PD-L1	miR-502-5p/HOXC8/SMAD3 axis promotes ICC proliferation and stabilizes PD-L1 via SPOP-mediated ubiquitination inhibition.	miR-502-5p	In vitro, in vivo, clinical	/	[172]

### 5.2. Circular RNA’s Role in Immunotherapy Resistance in Gastrointestinal Tumors

In gastrointestinal tumors, circRNA-mediated immunotherapy resistance is likewise marked by strong mechanistic convergence, particularly on the circRNA–miRNA–PD-L1/PD-1 axis. In gastric cancer (GC), circDLG1 is upregulated in distal metastases and in anti-PD-1-treated tissues, where it acts as an miR-141-3p sponge to increase CXCL12 expression and promote tumor progression and anti-PD-1 resistance [173] (Table 2). circcube3 binds miR-744-5p and affects SLC7A5/PD-L1 signaling, thereby reducing the anti-tumor activity of PD-L1 therapy [174] (Table 2). circ_0073453 promotes PD-L1 expression in GC cells by regulating IL-8 secretion from GC-MSCs through the circ_0073453/miR-146a-5p/IL-8 axis and thereby contributes to resistance to CD8+ T-cell killing [175] (Table 2). circRHBDD1 is elevated in GC and enhances PD-L1 expression by stabilizing IGF2BP2 while also reducing CD8+ T-cell infiltration; importantly, circRHBDD1 siRNA delivered by PLGA-PEG nanoparticles combined with anti-PD-L1 therapy has shown favorable in vivo efficacy [176] (Table 2). Exosomal circMAN1A2 (hsa_circ_0000118), abundant in GC-derived exosomes, suppresses T-cell anti-tumor activity by inhibiting FBXW11-mediated SFPQ degradation [177] (Table 2). Collectively, these findings indicate that GC-associated circRNAs primarily converge on checkpoint upregulation, T-cell dysfunction, and stromal cell-mediated immunosuppression.

In esophageal cancer, circ-VIM promotes immune escape through the miR-124/PD-L1 axis, and silencing circ-VIM synergizes with sevoflurane to suppress immune evasion and malignant phenotypes [178] (Table 2). circNF1 is associated with anti-PD-L1 immunotherapy response in esophageal squamous cell carcinoma and promotes immune escape through dual regulation of PD-L1: first, by activating the IL-6/JAK-STAT3 pathway, and second, by enhancing USP7-mediated PD-L1 stability via ANXA1 [179] (Table 2). These observations indicate that circRNAs can regulate both transcriptional induction and post-translational stabilization of immune checkpoints.

In colorectal cancer (CRC), circQSOX1 promotes glycolysis, immune escape, and resistance to anti-CTLA-4 therapy through the miR-326/miR-330-5p/PGAM1 axis [180] (Table 2). Hsa_circ_0136666 upregulates PD-L1 by sponging miR-497 and promotes Treg activation and immune escape [181] (Table 2). circ_0089761 and circ_0007422 also enhance PD-L1 expression through miR-27b-3p and miR-1256, respectively [182,183] (Table 2). In contrast, circPHLPP2 promotes tumor growth and anti-PD-1 resistance by binding ILF3 and regulating IL36γ transcription, thereby suppressing NK-cell infiltration and effector cytokine production [184] (Table 2). Exosomal circPOLQ activates the IL-10/STAT3 axis and promotes M2 macrophage polarization, reinforcing an immunosuppressive microenvironment [185] (Table 2). circNCOA3 is overexpressed in drug-resistant CRC and enhances proliferation, invasion, CD8+ T-cell function, and sensitivity to PD-1 therapy by regulating CXCL1 via miR-203aa-3p.1 [186] (Table 2). circRNF216 acts as a tumor suppressor in CRC; by functioning as a ceRNA for miR-576-5p, it increases ZC3H12C expression, promotes CD8+ T-cell infiltration, and inhibits tumor progression [187] (Table 2). CircMVP upregulates β-catenin and further increases B7-H3 expression, and suppression of circMVP improves anti-B7-H3 immunotherapy efficacy in vitro and in vivo [188] (Table 2). These studies indicate that CRC-associated circRNAs influence not only PD-1/PD-L1 but also other checkpoint pathways such as CTLA-4 and B7-H3.

In pancreatic cancer, hsa_circ_0046523 promotes CD8+ T-cell apoptosis and exhaustion, increases IL-10 and TGF-β secretion, reduces IFN-γ and IL-2, and upregulates PD-L1 through miR-148a-3p [189] (Table 2). CircRNA_102049 inhibits pancreatic cancer progression by upregulating CD80 via miR-455-3p, indicating that some circRNAs may enhance anti-tumor immunity rather than promote immune escape [190] (Table 2). In addition, circ_0001947 encapsulated in small extracellular vesicles promotes gastric cancer progression and anti-PD-1 resistance by modulating CD8+ T-cell exhaustion through CD39-related pathways [191].

Overall, gastrointestinal tumor-associated circRNAs converge on a limited number of immune-resistance modules, including PD-L1 upregulation, T-cell exhaustion, Treg induction, NK-cell suppression, M2 macrophage polarization, and metabolic rewiring. This redundancy suggests that many circRNAs may be functionally overlapping at the level of downstream effectors, despite differences in sequence or tumor context. Accordingly, translational value is likely to be greatest for exosomal or plasma-accessible circRNAs with tumor specificity and non-redundant positioning within the resistance network.

**Table 2:** The target and mechanism of circular RNA in immune therapy resistance of gastrointestinal tumors.

circRNA	Cancer Type	Immune Checkpoints	Altered Mechanism	Molecular Targets	Study Model	Drug	References
circDLG1	GC	PD-1	circDLG1 can act as an miRNA sponge to increase the expression of CXCL12.	miR-141–3p	In vitro, in vivo, clinical		[173]
circcube3	GC	PD-L1	Targeting SLC7A5 regulates PD-L1 to affect the immune escape of gastric cancer.	miR-744–5p	In vitro, in vivo		[174]
circ_0073453	GC	PD-L1	Resistance to killing by cytotoxic CD8^+^ T-cells.	miR-146a–5p	In vitro, clinical		[175]
circ-VIM	EC	PD-L1	The Ras/ERK signaling pathway was inactivated.	miR-124	In vitro, in vivo		[178]
circQSOX1	CRC	CTLA-4	It regulates glycolysis and promotes immune escape in CRC cells.	miR-326/miR-330-5p	In vitro, in vivo, clinical		[180]
circ_0136666	CRC	PD-L1	Stimulation of Treq cells.	miR-497	In vitro, in vivo		[181]
hsa_circ_0046523	PBMC	PD-L1	Apoptosis and exhaustion of CD8^+^ T-cells.	miR-148a–3p	In vitro, clinical		[189]
circRNA_102049	PBMC	PD-L1	The expression of CD80 was upregulated.	miR-455–3p	In vitro, clinical		[190]
circ_0001947	GC	PD-1	The upregulation of CD39 can facilitate CD8^+^ T-cell exhaustion.	miR-661/miR-671-5p	In vitro, clinical	/	[191]
circRHBDD1	GC	PD-L1	It enhances PD-L1 expression and impeds the infiltration of CD8^+^ T-cells.	IGF2BP2	In vitro, in vivo, clinical	/	[176]
circMAN1A2	GC	/	CircMAN1A2 inhibits T-cell anti-tumor activity by suppressing T-cell receptor signaling.	T-cell receptor signaling.	In vitro, clinical	/	[177]
circNF1	ESCC	PD-L1	IL-6-mediated JAK-STAT3 activation enhances p-STAT3 binding and USP7-dependent PD-L1 stabilization.	IL-6, ANXA1	In vitro, in vivo, clinical	/	[179]
circ_0089761	CRC	PD-L1	The expression of PD-L1 was upregulated.	miR-27b-3p	In vitro, in vivo		[182]
circ_0007422	CRC	PD-L1	The expression of PD-L1 was upregulated.	miR-1256	In vitro, in vivo		[183]
circPHLPP2	CRC	PD-1	The infiltration of NK-cells and the production of granzyme B and interferon-gamma (IFN-γ) are suppressed.	ILF3	In vitro, in vivo, clinical	/	[184]
circPOLQ	CRC	/	Targeting miR-379-3p to active IL-10/STAT3 axis for promoting M2 macrophage polarization.	miR-379-3p	In vitro, in vivo, clinical	/	[185]
circNCOA3	CRC	PD-1	Through the regulation of CXCL1 levels by miR-203aa-3p.1, sensitivity to PD-1 antibody therapy is enhanced.	miR-203aa-3p.1	In vitro, in vivo, clinical		[186]
circRNF216	CRC	/	circRNF216/miR-576-5p/ZC3H12C pathway was activated to increase CD8^+^ T-cell infiltration.	miR-576-5p	In vitro, clinical		[187]
circMVP	CRC	B7-H3	The expression of β-catenin is increased, leading to a subsequent upregulation of B7-H3.	β-catenin	In vitro, in vivo		[188]

### 5.3. Circular RNA’s Role in Immunotherapy Resistance in Lung Tumors

In lung tumors, circRNA-mediated immunotherapy resistance is driven by recurrent and highly convergent mechanisms, particularly the circRNA–miRNA–PD-L1/PD-1 axis, exosomal immune remodeling, and suppression of T-cell recruitment or effector function. In non-small cell lung cancer (NSCLC), circ-CPA4 is highly expressed and promotes tumor growth, stemness, drug resistance, and immune escape by regulating the let-7 miRNA/PD-L1 axis and inactivating CD8+ T-cells in the tumor immune microenvironment [192] (Table 3). hsa_circ_0003222 is also upregulated in NSCLC and lung cancer stem cells; its silencing reduces proliferation, migration, invasion, stemness, and resistance to anti-PD-L1 therapy by relieving miR-527-mediated repression of PHF21B and downstream β-catenin signaling [193] (Table 3). circ_CELF1 similarly promotes NSCLC progression and immunotherapy resistance through the miR-491-5p/EGFR axis [194] (Table 3). These studies indicate that distinct circRNAs often converge on shared outputs such as PD-L1 expression, EGFR signaling, and stemness-associated immune escape.

A second theme is exosomal circRNA-mediated crosstalk in the tumor microenvironment. Hypoxic extracellular vesicle-derived circPLEKHM1 enhances the interaction between PABPC1 and eIF4G, promotes OSMR translation, and polarizes macrophages toward the M2 phenotype, thereby facilitating metastasis and immune suppression [195] (Table 3). circUSP7, another cancer cell-derived exosomal circRNA, induces CD8+ T-cell dysfunction and anti-PD-1 resistance by regulating the miR-934/SHP2 axis [196] (Table 3). Similarly, exosomal circZNF451 restrains anti-PD-1 treatment in lung adenocarcinoma by polarizing macrophages through a TRIM56/FXR1/IRF4-related axis [197] (Table 3). These findings suggest that exosomal circRNAs are active mediators of immune suppression rather than passive byproducts.

Metabolic rewiring is also an important component of immune evasion in lung cancer. circRUNX1 enhances glycolysis and lactate accumulation through the miR-145/HK2 pathway, promoting Treg proliferation and immune escape [198] (Table 3). circASCC3 elevates C5a levels by sponging miR-432-5p and thereby limits anti-tumor immunity in NSCLC [199] (Table 3). These observations support the view that circRNAs remodel the metabolic and inflammatory landscape of the tumor microenvironment and thereby indirectly reduce immunotherapy responsiveness.

At the checkpoint level, hsa_circ_0000190 promotes tumorigenesis and immune evasion by upregulating soluble PD-L1 (sPD-L1), which interferes with anti-PD-L1 antibody efficacy and T-cell activation [200] (Table 3). circ_001678 promotes immune escape through the miR-326/ZEB1 axis and activates the PD-1/PD-L1 pathway, resulting in CD8+ T-cell apoptosis [201] (Table 3). circ_0068252 is associated with poor prognosis and cisplatin resistance and contributes to immune escape by upregulating PD-L1 through miR-1304-5p [202] (Table 3). hsa_circ_0020714 is highly expressed in NSCLC and is associated with poor prognosis and anti-PD-1 resistance via the miR-30a-5p/SOX4 axis [203] (Table 3). In addition, circ-HSP90A promotes NSCLC growth, stemness, and immune evasion through STAT3 signaling and PD-1/PD-L1 checkpoint regulation [204] (Table 3), while circENTPD7 and circ_0101675 upregulate PD-L1 via the IGF2BP2/PD-L1 axis or the miR-607/PDL1 axis, respectively [205,206] (Table 3). circCHST15 also promotes immune escape by targeting miR-155-5p and miR-194-5p and increasing PD-L1 expression [207] (Table 3).

Beyond PD-L1, lung cancer circRNAs regulate additional immune pathways and antigen-recognition programs. circFGFR1 promotes anti-PD-1 resistance by sponging miR-381-3p and increasing CXCR4 expression [208] (Table 3). circFNDC3B interacts with TFII-I to inhibit STAT1-mediated transcription of CXCL10 and CXCL11, thereby reducing CD8+ T-cell infiltration [209] (Table 3). hsa-circRNA-002178 upregulates PD-L1 in lung adenocarcinoma by sponging miR-34 and promoting T-cell exhaustion [210] (Table 3). circHMGB2 enhances immunosuppression and anti-PD-1 resistance in LUAD and lung squamous cell carcinoma by regulating the miR-181a-5p/CARM1 axis and inactivating type I interferon responses [211] (Table 3). By contrast, circMAPK1 enhances CD8+ T-cell infiltration by stabilizing CCL5 via IGF2BP1 and may improve response to immunotherapy [212] (Table 3).

Taken together, lung tumor-associated circRNAs converge on a limited set of downstream effectors, including PD-L1, EGFR, STAT3, CXCR4, C5a, SHP2, type I interferon signaling, and chemokine-mediated T-cell recruitment. This convergence indicates substantial mechanistic redundancy: multiple circRNAs may generate similar immune phenotypes through different upstream triggers. From a translational perspective, circRNAs with strong tumor specificity, exosomal detectability, or non-redundant positioning in immune networks are likely to be more feasible as biomarkers or combi-nation-therapy targets than as stand-alone interventions. Plasma circRNA profiling may also be useful for response prediction in EGFR-mutant NSCLC treated with gefitinib [192,193]. (Table 3).

**Table 3:** The target and mechanism of circular RNA in immune therapy resistance of lung tumors.

circRNA	Cancer Type	Immune Checkpoints	Altered Mechanism	Molecular Targets	Study Model	Drug	References
Circ-CPA4	NSCLC	PD-L1	Growth, migration, stemness, and drug resistance of inactivated CD8^+^ T-cells in the tumor immune microenvironment.	let-7 miRNA	In vitro, clinical		[192]
hsa_circ_0003222	NSCLC	PD-L1	The important role of hsa_circ_000322 in the stem cell-like characteristics of NSCLC cells.	miR-527	In vitro, in vivo, clinical		[193]
circ_CELF1	NSCLC	/	Increasing the expression of miR-491-5p target gene EGFR ultimately promotes the progression of NSCLC.	miR-491-5p/EGFR	In vitro, clinical		[194]
hsa_circ_0000190	NSCLC	PD-L1	Upregulation of sPD-L1 expression promotes the tumorigenesis and immune escape of NSCLC.	/	In vitro, clinical		[195]
circ_001678	NSCLC	PD-1/PD-L1	It also promoted the apoptosis of CD8^+^ T-cells.	miR-326/ZEB1	In vitro, in vivo		[196]
circ_0068252	NSCLC	PD-L1	It is involved in the regulation of cisplatin resistance and immune escape in NSCLC cells.	miR-1304-5p/PD-L1	In vitro, clinical		[197]
hsa_circ_0020714	NSCLC	PD-L1	Enhanced SOX4 expression.	miR-30a-5p	In vitro, clinical		[198]
circASCC3	NSCLC	PD-1	It can upregulate the level of complement C5a, promote the progression of NSCLC and immune dysfunction.	miR-432-5p	In vitro, clinical		[199]
circ-HSP90A	NSCLC	STAT3, PD-1/PD-L1	Regulation of STAT3 signaling pathway and programmed death 1 (PD-1)/PD-L1 checkpoint promotes the growth of NSCLC cells.	miR-424-5p	In vitro, in vivo		[200]
has-circRNA-002178	LUAD	PD-L1	T-cell exhaustion was induced.	miR-34	In vitro, clinical		[201]
circUSP7	NSCLC	PD-1	Inhibition of CD8^+^ T-cells, secretion of IFN-γ, TNF-α, granzyme b and perforin by CD8^+^ T-cells.	miR-934/SHP2	In vitro, in vivo, clinical		[202]
CircFGFR1	NSCLC	PD-1	The expression of C-X-C motif chemokine receptor 4 (CXCR4), the target gene of miR-381-3p, was upregulated.	miR-381-3p/CXCR4	In vitro, in vivo		[203]
CircZNF451	NSCLC	PD-1	Induction of macrophage polarization to reshape the tumor immune microenvironment.	FXR1- ELF4-IRF4	In vitro, in vivo, clinical		[204]
CircCHST15	NSCLC	PD-1/PD-L1	Immune escape of lung cancer cells.	miR-194-5p	In vitro, in vivo, clinical		[205]
circHMGB2	LUAD	PD-1	Inactivation of the type 1 interferon response.	miR-181a-5p/CARM1	In vitro, in vivo, clinical		[206]
circPLEKHM1	NSCLC	/	Polarizing macrophages towards the M2 phenotype.	PABPC1/eIF4G	In vitro, in vivo	/	[207]
circRUNX1	NSCLC	/	The proliferation of Treg cells.	miR-145	In vitro, in vivo	/	[208]
circFNDC3B	NSCLC	/	Downregulation of CD8^+^ T-cells.	TFII-I	In vitro, in vivo	/	[209]
circENTPD7	NSCLC	PD-L1	Promoting the expression of IGF2BP2 to upregulate PD-L1.	IGF2BP2	In vitro		[210]
circ_0101675	NSCLC	PD-L1	Upregulation of PD-L1 levels.	miR-607	In vitro, in vivo		[211]
circMAPK1	LUAD	/	Connecting IGF2BP1 to stabilizers CCL5 to recruit CD8^+^ cells.	IGF2BP1	In vitro, in vivo		[212]

### 5.4. Circular RNA’s Role in Immunotherapy Resistance in Urological Tumors

In urological tumors, circRNA-mediated immunotherapy resistance is shaped by both immune exclusion and metabolic suppression, with a major emphasis on CD8+ T-cell function. In bladder cancer (BCa), circMGA acts as a tumor-suppressive circRNA that enhances CD8+ T-cell chemoattraction and synergizes with anti-PD-1 therapy. Mechanistically, circMGA stabilizes CCL5 mRNA by interacting with HNRNPL, and HNRNPL in turn stabilizes circMGA, forming a positive feedback loop that supports anti-tumor immunity [213] (Table 4). Similarly, Hnrnpl-induced circFAM13B suppresses glycolysis and acidic tumor microenvironment formation through the circFAM13B/IGF2BP1/PKM2 cascade, thereby reducing immune escape and improving sensitivity to PD-1 antibodies [214] (Table 4).

By contrast, several circRNAs in BCa promote immune resistance through myeloid reprogramming and checkpoint activation. Bladder-cancer-derived exosomal circRNA_0013936 enhances FATP2 expression by targeting JAK2 via miR-320a and inhibits RIPK3 by sponging miR-301b-3p, thereby impairing CD8+ T-cell function and promoting suppressive immunity in PMN-MDSCs [215] (Table 4). HIF-1α-induced circFAM64A promotes BCa progression and inhibits CD8+ T-cells by sponging miR-149-5p and activating the IL-6/JAK/STAT pathway, which results in PD-L1 upregulation [216] (Table 4). circZNF609 also contributes to immune evasion and reduces immunotherapy sensitivity by enhancing fatty acid uptake through the IGF2BP2/CD36 pathway [217] (Table 4). These data suggest that circRNAs in bladder cancer can simultaneously regulate immune checkpoints, metabolic fitness, and myeloid-cell polarization.

In Wilms tumors, the circRNA landscape remains less well characterized, but transcriptomic studies indicate that differentially expressed circRNAs are embedded in networks related to cell-cycle control and immune response [218] (Table 4). The circR-NA-miRNA-mRNA ceRNA network derived from high-throughput RNA sequencing suggests that circRNAs may influence tumor immunobiology through broader regulatory modules rather than single pathways [218] (Table 4). This provides a basis for further mechanistic validation, particularly with respect to immune-cell recruitment and antigen presentation.

In renal cell carcinoma (RCC), circGRAMD4 represents a distinct mechanism of immune escape that directly affects antigen presentation. circGRAMD4 promotes autophagic degradation of MHC-I molecules by stabilizing NBR1 transcripts through interaction with RBM4, thereby impairing antigen presentation to cytotoxic T-cells and reducing CD8+ T-cell infiltration [219] (Table 4). Importantly, knockout of circGRAMD4 enhances anti-tumor immunity and improves response to immune checkpoint blockade [211]. This example extends circRNA function beyond checkpoint regulation to direct control of the antigen-presentation machinery.

Overall, urological tumor-associated circRNAs converge on glycolysis, fatty-acid metabolism, IL-6/JAK/STAT signaling, CD36-dependent lipid uptake, CCL5-mediated recruitment, and MHC-I antigen presentation. This suggests that the most clinically actionable circRNAs may be those located at non-redundant bottlenecks, such as anti-gen-presentation control or metabolic remodeling, although therapeutic feasibility will still depend on tumor type and immune context.

**Table 4:** The target and mechanism of circular RNA in immune therapy resistance of urological tumors.

circRNA	Cancer Type	Immune Checkpoints	Altered Mechanism	Molecular Targets	Study Model	Drug	References
circMGA	CUB	PD-1	Synergistic treatment between anti-PD-1 can significantly inhibit the growth of xenograft bladder cancer.	HNRNPL	In vitro, in vivo		[213]
circFAM13B	CUB	PD-1	Inhibition of BCa glycolysis and acidic TME.	IGF2BP1/PKM2	In vitro, in vivo		[214]
DE-circRs	WT	/	Multiple signaling pathways of DE-mRs are related to cell cycle and immune response.	/	In vitro, clinical		[215]
circRNA_0013936	BC	/	Through circRNA_0013936/miR-320a/JAK2 and circRNA_0013936/miR-301b-3p/CREB1pathway to inhibit CD8+ T-cell.	miR-320a/miR-301b	In vitro, clinical	/	[216]
circFAM64A (3)	BC	PD-L1	Triggering the JAK/STAT pathway and upregulating PD-L1 levels	miR-149-5p	In vitro, in vivo	/	[217]
circZNF609	BC	/	It can stabilize CD36 mRNA.	IGF2BP2	In vitro, in vivo	/	[218]
circGRAMD4	RCC	/	Decreasing the infiltration of CD8+ T-cells.	RBM4	In vitro, in vivo, clinical		[219]

### 5.5. Circular RNA’s Role in Immunotherapy Resistance in Gynecologic Tumors and Breast Cancer

In gynecologic tumors and breast cancer, circRNAs regulate immune resistance through checkpoint activation, immune-cell dysfunction, and epigenetic or translational control. In ovarian cancer (OC), hsa_circ_0025721 is associated with poor PD-1 responsiveness and CD8+ T-cell dysfunction, in part through the hsa-miR-4428/CXCL8 axis [220] (Table 5). circ-0001068 is enriched in exosomes from epithelial ovarian cancer (EOC) patients and is transferred to T-cells, where it induces PD-1 expression by acting as a ceRNA for miR-28-5p [221] (Table 5). circNFIX also promotes immune escape by sponging miR-647 and upregulating IL-6R, thereby activating the JAK/STAT3 pathway and increasing PD-L1 expression [222] (Table 5). These findings indicate that ovarian cancer circRNAs mainly converge on a checkpoint-centered suppressive program coupled with inflammatory signaling.

In breast cancer (BC), several circRNAs reinforce immunosuppression through MDSC recruitment, checkpoint activation, or interferon suppression. Circ-E-cadherin promotes recruitment and function of MDSCs, particularly PMN-MDSCs, by activating EGFR signaling and increasing CXCL8 transcription; importantly, targeting circ-E-cadherin improves anti-PD-1 efficacy [223] (Table 5). circGSK3β sponges miR-338-3p to release PRMT5, which promotes H3K4me3-mediated transcription of PD-L1 and facilitates immune evasion [224] (Table 5). circATAD2, an m6A-modified circRNA, stabilizes PD-L1 mRNA via IGF2BP3 and diminishes CD8+ T-cell anti-tumor surveillance [12] (Table 5). Similarly, circ_0001598 is upregulated in trastuzumab-resistant BC and promotes PD-L1-mediated immune escape by sponging miR-1184; restoring miR-1184 abolishes the oncogenic effects of circ_0001598, including trastuzumab resistance and escape from CD8+ T-cell killing [13] (Table 5).

circPVT1 illustrates that circRNAs can also regulate endocrine resistance and innate immune signaling. In ERα-positive breast cancer, circPVT1 promotes tumor growth and endocrine resistance by sponging miR-181a-2-3p and increasing ESR1 expression; in parallel, it interacts with MAVS to disrupt the RIG-I–MAVS complex, suppress type I IFN signaling, and weaken anti-tumor immunity [14] (Table 5). circTNK2 is overexpressed in tamoxifen-resistant breast cancer and contributes to both proliferation and immune escape. It interacts with SRSF1 to activate AKT/mTOR signaling, and its translated product, C-TNK2-487aa, inhibits NK-cell recruitment and enhances STAT3 phosphorylation, which suppresses CXCL10 expression [15] (Table 5). In contrast, circFAM53B encodes cryptic peptides that bind HLA and prime antigen-specific CD4+ and CD8+ T-cells, thereby eliciting anti-tumor immunity; higher expression of circFAM53B and its peptides is associated with increased CD8+ T-cell infiltration and improved survival in breast cancer and melanoma [16] (Table 5). Finally, circCFL1 promotes TNBC stemness and immune evasion by stabilizing mutant p53 through HDAC1/c-Myc signaling and by upregulating PD-L1 while reducing CD8+ T-cell infiltration [17] (Table 5).

Overall, the gynecologic and breast cancer literature highlights three recurrent modules: PD-L1-centered immune escape, interferon suppression, and myeloid/NK-cell remodeling. At the same time, it provides some of the clearest evidence that circRNAs can be exploited in multiple ways: as siRNA/ASO targets, as circulating biomarkers, or as antigen-encoding platforms [213,218,219,220]. However, circRNA function remains strongly context dependent, and a circRNA may be immune-suppressive in one setting but immunogenic in another. Therefore, the most promising translational candidates are likely to be those with clear compartment specificity, measurable circulation stability, and a direct mechanistic link to a non-redundant immune bottleneck.

**Table 5:** The target and mechanism of circular RNA in immune therapy resistance of gynecologic tumors and breast cancer.

circRNA	Cancer Type	Immune Checkpoints	Altered Mechanism	Molecular Targets	Study Model	Drug	References
hsa_circ_0025721	OC	PD-L1	It is related to the dysfunction of CD8^+^ T-cells.	hsa-miR-4428/CXCL8	In vitro, clinical		[220]
circ-0001068	OC	PD-L1	PDI expression was induced by competing endogenous RNA (ecRNA).	miR-28-5p	In vitro, clinical		[221]
circ_0001598	BC	PD-L1	All oncogenic effects leading to CD8^+^ T-cell killing were abolished.	miR-1184	In vitro, clinical		[222]
CircPYT1	BC	PD-L1	Inhibition of type I interferon (IFN) signaling and anti-tumor immunity.	RIGI-MAVS	In vitro, in vivo, clinical		[223]
circNFIX	OC	PD-L1	Through JAK/STAT3 pathway to increase PD-L1 levels.	miR-647	In vitro, in vivo	/	[224]
Circ-E-cadherin	BC	PD-1	Activating EGFR signaling and boosting CXCL8 transcription.	/	In vitro, in vivo		[12]
CircGSK3 β	BC	PD-L1	ThroughCircGSK3 β/miR-338-3p/PRMT5/H3K4me3.	miR-338-3p	In vitro, in vivo		[13]
circATAD2	BC	PD-L1	PD-L1mRNA was stabilized by bonding with circATAD2.	m6A PD-L1	In vitro, clinical		[14]
CircTNK2	BC	/	It interacts with STAT3and downregulate CXCL10.	STAT3	In vitro, in vivo, clinical		[15]
circFAM53B	BC	/	Encoding peptides to prime naive CD4^+^ and CD8^+^ T-cells in an antigen-specific manner.	HLA	In vitro, in vivo, clinical		[16]
CircCFL1	BC	PD-L1	Upregulation of PD-L1 and reducing CD8^+^ T-cell infiltration.	HDAC1, c-Myc	In vitro, in vivo, clinical		[17]

### 5.6. circRNA’s Role in Brain Cancer Resistance

The unique immune-privileged status of the central nervous system presents formidable barriers to conventional immunotherapies, a challenge underscored by a recent trial in recurrent glioblastoma showing an objective response rate of only 10.4% with combined PD-1 blockade [27]. Emerging evidence highlights that circular RNAs (circRNAs) act as master drivers of this profound resistance by orchestrating the local immunosuppressive microenvironment. A primary mechanism of circRNA-mediated evasion involves the modulation of glioblastoma-associated microglia/macrophages (GAMs). Specific circRNAs actively promote the M2-like polarization of these brain-resident myeloid cells by sustaining ERK1/2 and MAPK signaling and driving the secretion of IL-6 and IL-10; notably, counteracting this macrophage state—such as through CSF1R inhibition with GW2580—is required to rescue MHC-II presentation and T-cell killing [28]. Beyond microenvironmental remodeling, circRNAs also govern tumor-intrinsic immune escape networks. They intersect with specific kinase cascades, such as the checkpoint kinase 2 (Chek2) pathway, to actively dampen STING signaling and antigen presentation, thereby shielding glioma cells from CD8+ T-cell cytotoxicity [29]. Ultimately, by simultaneously rewiring macrophage polarization and intrinsic kinase signaling, circRNAs establish a robust, dual-layered resistance barrier in brain cancers.

### 5.7. Circular RNA’s Role in Immunotherapy Resistance in Other Tumors

In other tumor types, circRNA-related immunotherapy resistance has been described less extensively, but the available evidence again points to recurrent functional themes rather than isolated events. In squamous cell carcinoma, circFAT1 mediates the relationship between cancer stemness and immune evasion by promoting STAT3 activation; importantly, circFAT1 knockdown enhances PD-1 blockade efficacy by increasing CD8+ T-cell infiltration [30] (Table 6). This suggests that circRNAs may influence both tumor-intrinsic stemness and the immune context simultaneously.

In oral squamous cell carcinoma (OSCC), circKRT1 regulates immune escape through the miR-495-3p/PD-L1 axis [31] (Table 6). By sponging miR-495-3p, circKRT1 relieves repression of PD-L1 and thereby promotes tumor progression and immune evasion. This reinforces the broader theme that PD-L1 upregulation is one of the most recurrent outputs of circRNA-mediated resistance across cancer types.

A particularly interesting case is circBART2.2, an Epstein–Barr virus (EBV)-encoded circRNA that is highly expressed in nasopharyngeal carcinoma (NPC). circBART2.2 interacts with the helicase domain of RIG-I, activates IRF3 and NF-κB, and promotes PD-L1 transcription, thereby suppressing T-cell function and driving immune escape [32] (Table 6). This viral circRNA example broadens the field beyond host-derived circRNAs and suggests that pathogen-encoded circRNAs may also contribute to immune evasion through innate immune sensing pathways.

Although the number of studies in these tumor types remains limited, the available data indicate that circRNAs converge on checkpoint regulation, STAT3 signaling, and T-cell suppression, whereas viral circRNAs may additionally exploit innate immune sensors such as RIG-I. These observations should be interpreted cautiously, but they support the view that circRNA-mediated immune escape is a generalizable feature of tumor biology rather than a phenomenon restricted to selected cancers.

**Table 6:** The target and mechanism of circular RNA in immune therapy resistance of other tumors.

circRNA	Cancer Type	Immune Checkpoints	Altered Mechanism	Molecular Targets	Study Model	Drug	References
circFAT1	Squamous cell carcinoma	PD-1/STAT3	The infiltration of CD8^+^ cells into the tumor.	/	In vitro, in vivo		[30]
CircKRT1	OSCC	PD-L1	Promotes immune evasion.	miR-495-3p	In vitro, in vivo		[31]
circBART2.2	EBV	PD-L1	Activation of transcription factors IRF and NF-κ. B promotes the transcription of PD-L1.	RIG-I	In vitro, in vivo, clinical		[32]

### 5.8. Limitations and Translational Barriers

Despite rapid progress, several issues continue to limit the clinical translation of circRNA biology. First, circRNA function is highly context dependent: the same circRNA may act as an immune suppressor in one tumor type but have neutral or even immune-activating effects in another. This context dependence complicates target selection and makes single-circRNA approaches less likely to succeed across heterogeneous patient populations. Second, many circRNAs converge on the same downstream nodes, such as PD-L1, STAT3, type I interferon signaling, or myeloid-cell polarization, raising the possibility of substantial functional redundancy. In this setting, inhibition of a single circRNA may provide only partial benefit unless it occupies a non-redundant position in the regulatory network [33,34].

Third, delivery remains a major obstacle. Although circRNAs are stable and therefore attractive as circulating biomarkers, efficient and tissue-specific delivery of circRNA-targeted agents, such as siRNAs, antisense oligonucleotides, or CRISPR-based systems, remains challenging. Current nanoparticle and lipid-based platforms require further optimization with regard to tumor selectivity, endosomal escape, and off-target toxicity [35,36]. Fourth, the field still lacks standardized validation criteria. Robust circRNA studies should ideally combine RNase R resistance assays, junction-specific sequencing, and functional perturbation experiments; however, not all published reports meet these standards, which may contribute to reproducibility concerns [37,38]. While clinical cohort studies highlight strong observational correlations between circRNA expression and resistance [39,40,41]. definitive mechanistic causation is primarily established through in vivo models such as tumor xenografts [206,211].

From a translational perspective, the most feasible near-term applications may be the use of exosomal or plasma circRNAs as non-invasive biomarkers rather than direct therapeutic inhibition of every resistance-associated circRNA. In parallel, circRNAs may be more clinically useful when incorporated into combination strategies, such as circRNA silencing plus checkpoint blockade, metabolic inhibition, or myeloid reprogramming. The emerging concept of tumor-specific circRNA vaccines is also promising, since circRNAs can serve as stable antigen-encoding platforms and may encode cryptic peptides capable of eliciting anti-tumor immunity [46,47,220]. Nevertheless, these approaches remain at an early stage and require rigorous prospective validation, including pharmacokinetic assessment, safety evaluation, and testing in clinically relevant models. A concise overview of these translational opportunities and barriers is provided in Figure 3.

Overall, future work should move beyond descriptive cataloging toward mechanistically prioritized circRNA selection, network-level analysis, and well-designed translational studies. Only circRNAs that are both biologically non-redundant and technically tractable are likely to advance from mechanistic candidates to clinically actionable tools.

## 6. Conclusions and Perspective

CircRNAs have emerged as important regulators of cancer immunotherapy resistance through immune checkpoint regulation, exosomal intercellular communication, metabolic rewiring, myeloid-cell polarization, antigen-presentation defects, and suppression of T- and NK-cell function [42,43]. Across different malignancies, circRNAs appear to act not merely as biomarkers, but as active upstream regulators that can reshape tumor cell-intrinsic programs and remodel the tumor microenvironment. In particular, circRNA-mediated effects on checkpoint signaling, exosomal transfer, and intercellular communication within the TME are increasingly recognized as central mechanisms of immune escape [44].

Although the number of circRNAs reported in cancer continues to increase, future work should move beyond descriptive cataloging and focus on circRNAs that occupy non-redundant positions within resistance networks. CircRNAs linked to key nodes such as PD-L1/PD-1, type I interferon signaling, adenosine metabolism, antigen presentation, and chemokine-mediated immune exclusion may be especially relevant for translational development. To improve mechanistic rigor, standardized validation remains essential, including RNase R resistance assays and backsplice junction sequencing [45,46]. Prospective clinical studies will also be required to determine whether circRNAs can reliably predict immunotherapy response, resistance, and prognosis across tumor types [47,48].

In parallel, circRNAs may be more useful clinically when incorporated into combination strategies rather than as stand-alone targets. Preclinical studies have already shown that circRNA-targeted interventions can enhance the efficacy of immunotherapy or other anticancer treatments, supporting the feasibility of such combined approaches [49]. In addition, the emerging concept of tumor-specific circRNA-based vaccines and circRNA-encoded cryptic peptides offers an intriguing new direction for cancer immunotherapy, because circRNAs can generate immunogenic peptides capable of eliciting anti-tumor immunity [50]. However, the clinical feasibility of these approaches will depend on rigorous preclinical validation, safety assessment, and careful evaluation of immunogenicity and off-target effects.

Overall, circRNAs represent a promising but still early-stage class of molecules for overcoming cancer immunotherapy resistance. Future progress will depend on moving from association-based observations to mechanistically prioritized and clinically grounded translational research. In this context, a better understanding of how circRNAs coordinate multi-cell interactions within the tumor microenvironment may further reveal actionable vulnerabilities and support the development of more effective combination therapies.

## Data Availability

No new data were created or analyzed in this study. Data sharing is not applicable to this article.

## Abbreviations

ASO	antisense oligonucleotide
B2M	β-2-microglobulin
BC	breast cancer
BCa	bladder cancer
ceRNA	competing endogenous RNA
circRNAs	circular RNAs
CRC	colorectal cancer
CSC	cancer stem cell
ctDNA	circulating tumor DNA detection
CXCL12	C-X-C motif chemokine ligand 12
DC	dendritic cell
DPP4	dipeptidyl peptidase 4
EBV	Epstein–Barr virus
EGFR	epidermal growth factor receptor
EMT	epithelial–mesenchymal transition
ER	estrogen-receptor
FDA	Food and Drug Administration
GAL-8	galectin-8
GSEA	gene set enrichment analysis
HCC	hepatocellular carcinoma
HLA	human leukocyte antigen
HSC	hepatic stellate cell
ICB	immune checkpoint blockers
IFN-γ	interferon-gamma
IL-8	interleukin-8
IRES	internal ribosome entry site
JAK	janus kinase
LncRNA	long non-coding RNA
LUAD	lung adenocarcinoma
MAPK	mitogen-activated protein kinase
MDR	multidrug resistance
MDSCs	myeloid-derived suppressor cells
miRNA	microRNA
MYC	myelocytomatosis oncogene
NK	natural killer
NSCLC	non-small cell lung cancer
OSCC	oral squamous cell carcinoma
OSMR	oncostatin M receptor
PD-1	programmed cell death protein 1
PD-L1	programmed cell death ligand 1
PI3K	phosphoinositide 3-kinase
PTEN	phosphatase and tensin homolog
RCC	renal cell carcinoma
SLC7A5	solute carrier family 7 member 5
TAMs	tumor-associated macrophages
TFII-I	transcription factor II-I
TGF-β	transforming growth factor-β
TME	tumor microenvironment
TNBC	triple negative breast cancer
Treg	regulatory T
USP7	ubiquitin specific peptidase 7
VEGF	vascular endothelial growth factor
WT	Wilms tumor
